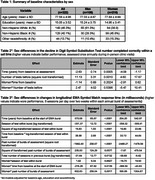# Sex differences in rates of cognitive decline based on conventional and ecological momentary assessments and accounting for practice effects

**DOI:** 10.1002/alz70857_107488

**Published:** 2025-12-26

**Authors:** Cuiling Wang, Qi Gao, Katherine H Chang, Mindy J. Katz, Carol A. Derby, Laura A. Rabin, Richard B. Lipton, Angel Garcia De La Garza

**Affiliations:** ^1^ Department of Epidemiology and Population Health, Department of Neurology, Albert Einstein School of Medicine, New York, USA, Bronx, NY, USA; ^2^ Albert Einstein College of Medicine, Bronx, NY, USA; ^3^ The Graduate Center, CUNY, Manhattan, NY, USA; ^4^ Queens College, CUNY, Flushing, NY, USA; ^5^ Department of Neurology, and Department of Epidemiology and Population Health, Albert Einstein College of Medicine, Bronx, NY, USA; ^6^ Brooklyn College of the City University of New York, Brooklyn, NY, USA; ^7^ The Graduate Center, CUNY, New York, NY, USA; ^8^ Department of Neurology, Albert Einstein College of Medicine, Bronx, NY, USA; ^9^ Montefiore Medical Center, Bronx, NY, USA

## Abstract

**Background:**

Despite evidence for sex differences in cognitive performance and risk of developing Alzheimer's disease and related dementias, few studies have explored sex differences in rates of cognitive decline. Examining rates of decline using longitudinal data can critically further our understanding of healthy versus abnormal cognitive aging; however, these studies are often complicated by practice effects. Smartphone‐based ecological momentary assessments (EMA) may provide a unique opportunity to address this concern by facilitating short‐term intensive measures of cognitive performance, multiple times/day over multiple days in real‐world settings.

**Method:**

The Einstein Aging Study assessed processing speed in diverse community‐dwelling older adults over 6 years using the WAIS‐III Digit Symbol Substitution Test (DSST) and a smartphone‐based EMA Symbol Match (SM) test. DSST was assessed once annually during in‐person clinic visits, and EMA SM was assessed via a burst protocol (i.e., 6 times/day over two weeks), repeated annually. Multi‐level linear mixed effects models were used to evaluate sex differences in age‐related cognitive decline while accounting for practice effects from exposure to repeat testing. Models were adjusted for covariates including age at baseline, years of education, and race/ethnicity.

**Result:**

Among 322 non‐demented older adults (77.6±5.0 years; 67.1% Female; 46.3% non‐Hispanic White, 40.1% non‐Hispanic Black), up to 6 annual bursts of EMA (mean 3.2±1.7) were obtained during a mean of 2.8±1.9 years of follow‐up. Consistent with prior literature, women performed better at baseline on both DSST number completed correctly within a set time (Table 2) and EMA SM response time (Table 3) measures. There were significant rates of decline among men in DSST and EMA SM during follow‐up. For EMA SM median completion time, mean performance at baseline was not significantly different between men and women; although, women showed faster decline during follow‐up (difference 216±88 milliseconds/year, *p* = 0.014) and slower improvement from burst assessment exposure (*p* = 0.002).

**Conclusion:**

We observed significantly faster decline in processing speed among women compared with men for EMA‐based but not a conventional, paper‐and‐pencil test. After accounting for practice effects, smartphone‐based digital EMA may be more sensitive to cognitive change and offer novel opportunities to identify possible sex differences at early disease stages.